# A randomized trial of amifostine as a cytoprotective agent in patients receiving chemotherapy for small cell lung cancer

**DOI:** 10.1054/bjoc.2000.1539

**Published:** 2001-01-01

**Authors:** P W M Johnson, M F Muers, M D Peake, K M Poulter, E M Gurney, V Napp, P M Hepburn, J M Brown

**Affiliations:** ICRF Cancer Medicine Research Unit, St James's University Hospital, Beckett Street, Leeds, UK; Leeds General Infirmary, Great George Street, Leeds, LS1 3EX, UK; Pontefract General Infirmary, Friarwood Lane, Pontefract, WF8 1PL, UK; Northern and Yorkshire Clinical Trials and Research Unit, 17 Springfield Mount, Leeds, LS2 9NG, UK

**Keywords:** small cell lung cancer, chemotherapy, myelotoxicity, chemoprotection, amifostine, survival

## Abstract

A randomized trial was conducted to determine whether administration of Amifostine with chemotherapy for small cell lung cancer could decrease the toxicity. 84 patients with small cell lung cancer of favourable prognosis (limited disease, performance status 0–1; limited disease with performance status 2 but normal sodium and alkaline phosphatase, or extensive diseas with performance status 0–1, normal sodium and alkaline phosphatase) received treatment with Ifosfamide 3 g/m^2^intravenously, Carboplatin (Glomerular filtration rate + 25) ×6 mg intravenously, Etoposide 50 mg orally, twice daily, for 7 days, every 3 weeks. Patients were randomized to receive amifostine 740 mg/m^2^immediately prior to the intravenous drugs (*n* = 42) or to receive chemotherapy alone (*n* = 42). The two groups were similar with respect to baseline prognostic factors. There was no significant difference in the occurrence of grade III or IV neutropenia or thrombocytopenia between the two groups, nor in the response rate or overall survival, for which the median was 11 months in the chemotherapy only group and 14 months in the group treated with amifostine. This study has not shown a protective effect from the use of amifostine with this regimen and there does not appear to be any effect upon the efficacy of treatment. © 2001 Cancer Research Campaign http://www.bjcancer.com


*British Journal of Cancer* (2001) **84**(1), 19–24
© 2001 Cancer Research Campaign
doi: 10.1054/ bjoc.2000.1539, available online at http://www.idealibrary.com on IDEAL®

http://www.bjcancer.com

### A randomized trial of amifostine as a cytoprotective agent in patients receiving chemotherapy for small cell lung cancer

**PWM Johnson[1], MF Muers[2], MD Peake[3], KM Poulter[4], EM Gurney[4], V Napp[4], PM Hepburn[4] and JM Brown[4]**

[1]ICRF Cancer Medicine Research Unit, St James's University Hospital, Beckett Street, Leeds, UK; [2]Leeds General Infirmary, Great George Street, Leeds, LS1 3EX, UK; [3]Pontefract General Infirmary, Friarwood Lane, Pontefract, WF8 1PL, UK; [4]Northern and Yorkshire Clinical Trials and Research Unit, 17 Springfield Mount, Leeds LS2 9NG, UK

**Summary** A randomized trial was conducted to determine whether administration of Amifostine with chemotherapy for small cell lung cancer could decrease the toxicity. 84 patients with small cell lung cancer of favourable prognosis (limited disease, performance status 0–1; limited disease with performance status 2 but normal sodium and alkaline phosphatase, or extensive diseas with performance status 0–1, normal sodium and alkaline phosphatase) received treatment with Ifosfamide 3 g/m$^2$ intravenously, Carboplatin (Glomerular filtration rate + 25) $\times$6 mg intravenously, Etoposide 50 mg orally, twice daily, for 7 days, every 3 weeks. Patients were randomized to receive amifostine 740 mg/m$^2$ immediately prior to the intravenous drugs ($n$ = 42) or to receive chemotherapy alone ($n$ = 42). The two groups were similar with respect to baseline prognostic factors. There was no significant difference in the occurrence of grade III or IV neutropenia or thrombocytopenia between the two groups, nor in the response rate or overall survival, for which the median was 11 months in the chemotherapy only group and 14 months in the group treated with amifostine. This study has not shown a protective effect from the use of amifostine with this regimen and there does not appear to be any effect upon the efficacy of treatment. © 2001 Cancer Research Campaign http://www.bjcancer.com

**Keywords:** small cell lung cancer; chemotherapy; myelotoxicity; chemoprotection; amifostine; survival

Combination chemotherapy in the management of early stage small cell lung cancer (SCLC) is recognized as beneficial, despite the low numbers of long-term cures achieved (Souhami and Law, 1990). Combination treatment yields a higher proportion of clinically complete responses than single agent therapy, and two-year survival rates of up to 30% have been reported from the use of ifosfamide, carboplatin and etoposide (ICE) with thoracic radiotherapy for patients with limited disease (Prendiville et al, 1994). There is some evidence from randomized trials that the dose intensity of treatment for SCLC may be important, with modest but definite improvements in survival seen following the use of growth factors to reduce the intervals between cycles of chemotherapy (Steward et al, 1998). An alternative strategy for the intensification of treatment is to use a cytoprotective agent to reduce normal tissue toxicity, which might in turn allow dose escalation.

Amifostine was originally identified as a radioprotective, but has also proved to have chemoprotective properties (van der Vijgh and Peters, 1994). The selectivity of its protective action in normal tissues rather than tumours is based upon the much higher concentrations of active metabolites found in normal cells, owing to differences in enzyme content (particularly alkaline phosphatases) (Yuhas, 1980). Once reduced to the active form, the drug acts as a free-radical scavenger and impairs DNA adduct formation by a variety of cytotoxic agents. Apart from in vitro and animal data confirming this action, there is data from a randomized trial in ovarian cancer in which patients received cisplatin and cyclophosphamide with or without amifostine (Glick et al, 1992). Those treated with amifostine experienced significantly less neutropenia, required fewer admissions to hospital for management of complications and were less likely to have treatment stopped prematurely owing to renal impairment. There was no difference in response rates or survival between the two arms.

In the light of these observations, this study was conducted to test the effect of amifostine in protecting against the myelosuppressive effects of a modified ICE chemotherapy regimen in patients with early small cell lung cancer. This regimen, comprising slightly attenuated doses of the cytotoxic drugs compared to the original study, had been tested in a phase II trial previously by the Yorkshire Thoracic Group (Shevlin et al, 1998). This had demonstrated a response rate in keeping with other chemotherapy regimens (83%, with 40% complete response) and a median survival among patients with good prognosis disease of 12.6 months (95% CI = 11.6–14.7 months). Despite the reduced drug dosages however, haematologic toxicities remained troublesome: 80% of patients experienced grade III/IV neutropenia and 60% grade III/IV thrombocytopenia. The purpose of the study was therefore to determine whether the delivery of this treatment could be made less toxic by the co-administration of a cytoprotective agent, with the intention of undertaking dose escalation subsequently if this proved to be effective.

### MATERIALS AND METHODS

### Patient eligibility and randomization

Patients were eligible for inclusion if they had microscopically confirmed SCLC with either limited or extensive disease. Patients with limited disease had WHO performance status 0 or 1, or 2 if both

*Received 19 April 2000*
*Revised 26 August 2000*
*Accepted 13 September 2000*

*Correspondence to:* V Napp the serum sodium and alkaline phosphatase levels were normal. Patients with extensive disease were included if their performance status was 0 or 1 and the sodium and alkaline phosphatase were normal. An upper age limit of 75 years was set, and those with significant renal impairment (creatinine > 150 μmol $1^{-1}$), hepatic impairment (bilirubin >50 μmol $l^{-1}$) or heart disease (NYHA grade III or worse) were excluded. Radiological staging investigations included chest X-ray and imaging of the abdomen by computed tomography or ultrasound. Computed tomographic scans of the brain, bone scintigraphy and marrow examinations were not required in the absence of indicative symptoms, signs or blood test abnormalities.

Following informed consent, patients were randomized 1:1 by telephone stratified by treatment centre. They were allocated treatment with either chemotherapy alone (C) or chemotherapy preceded by amifostine (A).

### Treatment

The chemotherapy comprised Carboplatin at a dose of 6× (Glomerular filtration rate +25) mg given over 1 hour, Ifosfamide 3 g/$m^2$ given as a 1 hour infusion with 3 g/$m^2$ Mesna, and Etoposide 50 mg b.d. orally for 7 days. Further Mesna was given following the Ifosfamide infusion: 1.8 g/$m^2$ was given by 8-hour intravenous infusion on the first cycle and, if this was well tolerated, 1.2 g was administered orally at 2 and 6 hours on subsequent cycles. Treatment was scheduled to be repeated every 21 days to a maximum of 6 cycles. In the Amifostine arm the chemotherapy was identical, but Amifostine 740 mg/$m^2$ was administered as a 10-min intravenous infusion immediately prior to the cytotoxics. Patients were treated lying down and their blood pressure monitored carefully during and after amifostine infusion in view of the known hypotensive effects of the drug. The infusion was interrupted if the systolic blood pressure dropped significantly or the patient became symptomatic, and normal saline was administered at 20 ml $min^{-1}$ until recovery. Patients received routine anti-emetic prophylaxis with 5HT-3 antagonists and dexamethasone, and were prescribed oral antibiotic prophylaxis to commence at day 8 of each cycle. Patients did not receive haemopoietic growth factors.

### Nadir counts

Following treatment the nadir blood count was measured at day 10–12 of each cycle. A further blood count was performed at day 21–22 and treatment continued provided the absolute neutrophil count was $>2 \times 10^9$ $l^{-1}$ and the platelet count $>100 \times 10^9$ $l^{-1}$. In cases where the counts were lower than this, treatment was delayed until recovery. In those cases where the nadir count showed neutrophils $<0.5 \times 10^9$ $l^{-1}$ or platelets $<25 \times 10^9$ $l^{-1}$, or where treatment was delayed due to incomplete recovery, all subsequent cycles were administered with 25% reduction of the doses of Ifosfamide and Carboplatin, and 6 days of oral etoposide instead of 7. Patients requiring more than one dose modification were withdrawn from the trial. Continuation with chemotherapy treatment alone was permitted at the clinician's discretion.

Patients with limited disease were recommended to receive consolidation thoracic radiotherapy 4 weeks after the last cycle of chemotherapy at a dose of 40 Gy in 15 fractions. No recommendation was made concerning prophylactic cranial irradiation.

### Assessments

Patients were followed up for two years from the completion date of chemotherapy or until death, whichever was sooner. Assessments were made prior to each cycle of treatment, at one month from the completion of treatment and 3 monthly thereafter. Tumour responses were assessed using standard WHO criteria (WHO, 1979). Assessment of toxicity was carried out by the treating clinician prior to each cycle of therapy and at the first follow-up visit. Patients completed daily diary cards from the first day of chemotherapy until 4 weeks after the last cycle of treatment. The cards were based upon those developed by the MRC Lung Cancer Working Party and required patients to record their grade of nausea, vomiting, difficulty in swallowing, activity, mood and overall condition using specified scales. The cards were also used to record the taking of etoposide, antiemetic and antibiotic medications.

### End points

The primary outcome measure was haematologic toxicity, specifically the occurrence of grade III/IV neutropenia or thrombocytopenia. Secondary endpoints were overall survival, response rate, non-haematological toxicities and patient-reported symptoms (i.e. nausea, vomiting, difficulty in swallowing, activity, mood and overall condition as recorded in the daily diary cards).

### Sample size

The study was designed to detect a 50% reduction of grade III/IV neutropenia or thrombocytopenia, taking as the anticipated levels the incidence of these in the previous phase II pilot study. Thus in order to detect a reduction of neutropenia from 80% to 40% or of thrombocytopenia from 60% to 30%, 84 patients were required for 80% power at the two-sided 5% significance level.

### Statistical analysis

Analyses were conducted on an intention-to-treat basis for all eligible randomized patients, except in the analyses of toxicity data, where patients were analysed according to the treatments they actually received.

The maximum severities of neutropenia and thrombocytopenia were calculated for each patient, over all cycles of treatment in the trial, and the proportions experiencing at least one episode of WHO grade III or IV toxicity were compared between the two treatment groups using the $\chi^2$ test at the 5% significance level. For each toxicity proportion, the corresponding 95% confidence interval was calculated. Where patients had missing data for toxicity, an additional analysis was performed assuming the worst case scenario (i.e. WHO grade IV toxicity). Analyses were compared to assess the potential impact of the missing data.

Within each treatment group, all symptoms of non-haematological toxicity were summarized independently as the number of patients who experienced at least one episode of WHO grade III or IV toxicity.

The assessments of response were summarized, after each cycle of chemotherapy, using the WHO criteria. The objective best response achieved over all cycles of chemotherapy was documented for each treatment group.

Survival was measured from the date of randomization until death from any cause. Patients still alive at the time of analysis

 © 2001 Cancer Research Campaign

(January 2000) had their survival time censored at the date of their last recorded follow-up visit. Survival estimates were calculated using the Kaplan-Meier method (Kaplan and Meier, 1958).

The compliance of patients completing diary cards was calculated on a card basis, by dividing the number of completed diary cards received by the number of diary cards expected. The percentage of patient days when each grade of symptom was experienced was calculated for each cycle using the diary card recordings for the first 21 days following chemotherapy (i.e. the planned length of the chemotherapy cycle).

All analyses were performed using SAS software version 6.12.

The protocol was approved by the local research ethical committee of each participating centre. Written informed consent was obtained from each patient before entry into the study.

### RESULTS

84 patients (42 in each group) were randomized between November 1996 and October 1998 from 16 Northern and Yorkshire centres and 1 Southern centre. The two groups were well matched at randomization for the known prognostic factors, except for serum albumin where more patients in A had a level below 35 g $l^{-1}$ (Table 1).

All the patients received treatment according to the randomization, except for one. This patient was randomized to A but suffered hypotension and was given the sixth cycle without amifostine due to safety concerns. The total amount of chemotherapy given, on study, was slightly lower in A, with 171 cycles in C and 141 cycles in A. The dose intensity of the chemotherapy was the same in both arms. Under the strict criteria for withdrawal following a second episode of myelosuppression, 30 (71%) patients in C and 23 (55%) patients in A were withdrawn from the study (Table 2). For the 30 patients in C, the median (range) number of chemotherapy cycles received, on study, was 3 (2 to 6) and for the 23 patients in A, the median (range) was 2 (2 to 6). Off study (i.e. after withdrawal for myelosuppression), the median (range) number of chemotherapy cycles received was 0.5 (0 to 4) for the patients in C and 1 (0 to 3) for the patients in A. The medians after withdrawal excluded 4 patients in C and 5 patients in A, as it was unknown whether they received further chemotherapy after follow-up.

Amifostine infusions were interrupted or the total dose reduced owing to side effects, principally hypotension, for some patients. 15 (36%) patients did not receive the full dose of amifostine on at least one occasion, and of 141 infusions given, 27 (19%) were at a reduced rate.

There was no statistically significant difference between the two arms in terms of the neutropenia or thrombocytopenia observed (Table 3). The overall incidence of at least one episode of grade III or IV neutropenia during treatment was 78% in C and 71% in A, and at least one episode of grade III or IV thrombocytopenia was 14% in C and 26% in A. When missing data was treated as the

**Table 1** Characteristics of randomized patients

| | | ICE (C) | ICE + amifostine (A) |
|---|---|---|---|
| Number of patients randomized | | 42 | 42 |
| Males: Females | | 24:18 | 28:14 |
| Median age (range) in years | | 64 (45–75) | 65 (43–74) |
| WHO performance status: | | | |
| | 0 | 16 (38%) | 20 (48%) |
| | 1 | 24 (57%) | 21 (50%) |
| | 2 | 2 (5%) | 1 (2%) |
| Disease extent: | | | |
| | Limited | 35 (83%) | 34 (81%) |
| | Extensive | 7 (17%) | 8 (19%) |
| Presence of metastases: | | | |
| | Present | 6 (15%) | 8 (19%) |
| | Absent | 35 (85%) | 34 (81%) |
| | Missing | 1 | 0 |
| *Biochemistry at randomization:* | | | |
| Sodium | <135 | 7 (18%) | 8 (19%) |
| (mmol/l) | ≥ 135 | 33 (83%) | 34 (81%) |
| | Missing[a] | 2 | 0 |
| Albumin | <35 | 3 (8%) | 6 (15%) |
| (g/l) | ≥35 | 37 (92%) | 35 (85%) |
| | Missing[a] | 2 | 1 |
| Alkaline phosphate | < 300 | 37 (93%) | 39 (95%) |
| (iu/l) | ≥ 300 | 3 (7%) | 2 (5%) |
| | Missing[a] | 2 | 1 |
| LDH | <450 | 13 (46%) | 7 (32%) |
| (iu/l) | ≥450 | 15 (54%) | 15 (68%) |
| | Missing[a] | 14 | 20 |
| NSE | <12.5 | 4 (17%) | 7 (32%) |
| | ≥12.5 | 20 (83%) | 15 (68%) |
| | Missing[a] | 18 | 20 |

[a]These results cannot be retrieved as they were not done at the time of blood assay.

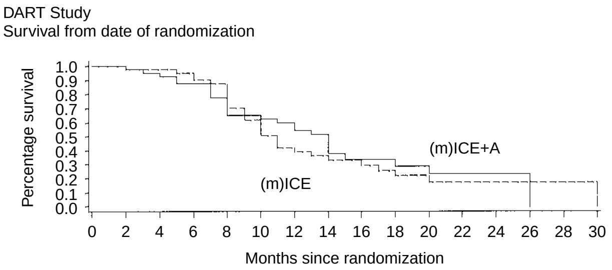

Number at risk

| | | | | | | | | | | | | | | | | |
|---|---|---|---|---|---|---|---|---|---|---|---|---|---|---|---|---|
| (m)ICE | 42 | 41 | 39 | 38 | 31 | 22 | 14 | 11 | 9 | 7 | 5 | 3 | 2 | 2 | 1 | 1 |
| (m)ICE+A | 42 | 41 | 38 | 35 | 31 | 26 | 22 | 15 | 7 | 7 | 5 | 4 | 3 | 1 | 0 | 0 |

**Figure 1**: Overall survival for the randomized treatment arms (Kaplan-Meier estimates)

**Table 2** Reasons for withdrawals

| | ICE (C) (*n* = 42) | ICE + amifostine (A) (*n* = 42) |
|---|---|---|
| Number of withdrawals | 33 (79%) | 35 (83%) |
| Reasons for withdrawal[a] | | |
| Evidence of tumour progression | 0 | 4 (10%) |
| Failure to respond after 2 cycles | 0 | 1 (2%) |
| Myelosuppression requiring second 25% dose reduction | 30 (71%) | 23 (55%) |
| Serious allergic reaction to amifostine | 0 | 1 (2%) |
| Patient develops need for palliative radiotherapy | 1 (2%) | 0 |
| Patient desires to be withdrawn | 2 (5%) | 7 (17%) |
| Other reasons[b] | 1 (2%) | 3 (7%) |

[a]Possible to have more than one reason for withdrawal. [b]Other reasons: ICE arm: Toxicity – severe paresthesia one side of body (may be stroke related); ICE + amifostine arm: Refused amifostine due to vomiting when first administered, Congestive cardiac failure and arterial fibrilation, Severe anaemia.

*© 2001 Cancer Research Campaign*

**Table 3** Proportion of patients experiencing at least one episode of WHO grade III or IV neutropenia and/or thrombocytopenia

| | ICE (C) | ICE + amifostine[a] (A) | Test statistic, df *P* value |
|---|---|---|---|
| Number of patients randomized | 42 | 42[b] | – |
| Grade III or IV neutropenia (%) | 33 (78%) | 30 (71%) | $\chi^2$ = 0.675, df = 1 |
| 95% C.I. | (66 91%) | (58, 85%) | *P* = 0.411 |
| Grade III or IV thrombo-cytopenia (%) | 6 (14%) | 11 (26%) | $\chi^2$ = 2.004, df = 1 |
| 95% C.I. | (4, 25%) | (13, 40%) | *P* = 0.157 |
| Grade III or IV neutropenia and/ or thrombocytopenia (%) | 33 (78%) | 30 (71%) | – |
| 95% C.I. | (66, 91%) | (58, 85%) | – |

95% CI = 95% Confidence interval for the proportion of patients with WHO grade 3 or 4 toxicity. [a]Chi-square test statistic, df, *P*-values quoted are unadjusted. [b]42 patients randomized to ICE and amifostine, but one patient did not receive amifostine for their last cycle. This patient had already experienced grade 3/4 toxicity before cycle 6.

**Table 4** Patient reported toxicity (percentage of patient days when moderate/severe grade of symptom was experienced)

| | | Cycle 1 | Cycle 2 | Cycle 3 | Cycle 4 | Cycle 5 | Cycle 6 |
|---|---|---|---|---|---|---|---|
| **Nausea** | C | **3.5%** | **3.4%** | **1.8%** | **1.2%** | **4.8%** | **4.8%** |
| Grades 3 & 4 | A | 3.0% | 1.8% | 2.4% | 2.1% | 1.6% | 0.8% |
| **Vomiting** | C | **2.4%** | **1.2%** | **1.0%** | **0.7%** | **0.3%** | **0** |
| Grades 3 & 4 | A | 0.9% | 0.6% | 0.6% | 1.0% | 0.8% | 1.6% |
| **Swallowing** | C | **2.4%** | **0.9%** | **0** | **0** | **0.5%** | **1.0%** |
| Grades 3, 4 & 5 | A | 2.9% | 0.3% | 0 | 0.3% | 0 | 0 |
| **Activity** | C | **8.0%** | **5.5%** | **7.1%** | **6.6%** | **5.0%** | **1.9%** |
| Grades 4 & 5 | A | 13.6% | 8.0% | 11.3% | 11.0% | 11.9% | 2.4% |
| **Mood** | C | **5.7%** | **3.4%** | **5.5%** | **4.7%** | **5.0%** | **3.1%** |
| Grades 4 & 5 | A | 5.9% | 4.1% | 4.4% | 5.3% | 3.2% | 1.6% |
| **Overall** | C | **5.8%** | **3.2%** | **5.8%** | **4.3%** | **4.8%** | **4.1%** |
| Grades 4 & 5 | A | 7.0% | 2.6% | 2.5% | 0.8% | 0.8% | 1.6% |

C = ICE alone; A = ICE + amifostine; Nausea grades 3 & 4 = moderate / severe; Vomiting grades 3 & 4 = sick 2 or more times per day; Swallowing grades 3, 4 & 5 = can swallow solids with difficulty / can't swallow solids / can't swallow liquids; Activity grades 4 & 5 = confined to home or hospital / confined to bed; Mood grades 4 & 5 = miserable / very miserable; Overall grades 4 & 5 = poor / very ill worst case scenario (i.e. WHO toxicity grade IV) there was no statistically significant difference between the two groups. Examining only cycle 1, to eliminate the potential confounding effects of dose reductions and withdrawals, shows a similar picture: 57% in C and 50% in A experienced grade III or IV neutropenia. There is some suggestion of an effect upon grade III or IV thrombocytopenia: in C the incidence was 5% and in A 17%.

There was no difference in the number of infectious episodes, bleeding, platelet transfusions or red blood cell transfusions between the two arms.

The non-haematologic toxicity, such as alopecia, gastrointestinal toxicity, renal impairment and neurotoxicity were equally common in both arms. However, grade III/IV nausea and vomiting showed a slight excess in the C arm: this occurred in 12 of 42 (29%) C patients compared to 3 of 42 (7%) A patients (95% CI for difference = 6% to 38%).

The objective best response rates were similar in both arms. Overall there were 38 patients (90%) in each arm who showed a complete or partial response at some point during their treatment.

In total 56 patients died: 29 in C and 27 in A. The overall survival was similar, however the median overall survival time was slightly higher in A: 14 months in A and 11 months in C (Figure 1). At 12 months since randomization overall survival in C was 42% and in A 60% (95% CI for difference = −3% to 39%).

The diary cards were effective as a means of gathering subjective toxic events to treatment. 74% of cards were returned, equally distributed between the two treatments, with good completion of the data items requested. The symptoms reported were similar, except for nausea and vomiting which showed a trend towards lesser severity in A, and overall condition which was rated higher in A, particularly following the second and subsequent cycles (Table 4). The effects of withdrawals were examined and it was found that these did not cause the estimates to be biased.

### DISCUSSION

The 90% objective response rate and median one-year overall survival in this study confirm the activity of the attenuated ICE regimen against small cell lung cancer of relatively favourable prognosis. These figures compare well with those reported from other recent trials of combination chemotherapy (Roth et al, 1992; Miles et al, 1994), although clearly a randomized study would be required to confirm the therapeutic equivalence of the lower dose regimen. Recent evidence suggests that small gains in survival may be achieved with intensification of the ACE regimen using growth factors to shorten the cycle length, and this benefit only became apparent when more than half the patients had died and comparison was made of the long survival groups (Thatcher et al, 2000). It would therefore be premature to conclude that the attenuated ICE regimen used in this study is truly as effective as more intensive chemotherapy, and further studies will be necessary to examine this.

This study has demonstrated the feasibility of administering a cytoprotective agent together with chemotherapy, although the full dose could not be given in all patients owing to toxicity. The use of amifostine does not appear to confer any protection upon the tumour itself. This was an important theoretical concern for such studies, particularly in the setting of SCLC where acquired chemoresistance is common. Although there was a small excess of early treatment failure in A, the cycle-by-cycle response rates in the two arms were comparable through the treatment, with no suggestion that responses in A rose after withdrawal from the study. Most importantly the overall survival was similar. Another concern regarding the use of amifostine was the possibility of increased side effects such as nausea and vomiting. In practice this was not problematic in A, and indeed there was a trend towards less nausea and vomiting. It is not clear why this should have been the case: the use of 5-$HT_3$ blocker anti-emetics was standardized in both arms and no difference was recorded in the adherence to the protocol in this respect. The finding of better scores for overall condition on the diary cards in A was unexpected given previously documented toxicity profile of the drug, but it is reassuring to know that it did not have any detrimental effect from the perspective of the patient. The slight excess of withdrawals at the request of patients in A (17% compared to 5%) was not apparently related to the side-effects of the amifostine. No calculation of statistical significance

© 2001 Cancer Research Campaign has been made for this data since this was not specified as a primary endpoint and is a post-hoc summary based on inspection of data. A post-hoc analysis is therefore liable to over-interpretation.

The principal end-point of the study was haematologic toxicity, and amifostine as used here does not appear to have exerted a significant effect, there is a slight increase in thrombocytopenia after the first cycle. Randomized studies of supportive therapy are complex to interpret over a prolonged course of treatment owing to the potential confounding effects of dose reductions and withdrawal of patients with excessive toxicity, and in this respect the data from the first cycle is the most reliable. However use of strict withdrawal criteria was a means to give a further indication of the comparative degree of myelosuppression as it might be relevant in practice, and here there was a trend in favour of the A arm.

Several reasons can be put forward to explain the lack of effect by amifostine in this trial compared to previous randomized studies which have demonstrated a reduction of myelosuppression. The chemotherapy used in this protocol may be less amenable to the cytoprotective effect of amifostine, and in particular the prolonged administration of oral etoposide is a likely confounding factor. The etoposide can be expected to exert a myelotoxic effect for considerably longer than the duration of action of the amifostine, which has an $\alpha$ half life of less than 1 minute and $\beta$ half-life of 9.5 minutes in serum, although the intracellular effect is likely to be much more prolonged (Yuhas, 1980). In designing the study it was anticipated that if there was a significant effect upon the toxicity produced by ifosfamide and carboplatin administered immediately after the amifostine this might result in less overall myelosuppression. However it is possible that the dominant effect upon the bone marrow is exerted by the etoposide for which the amifostine was not effective. Given the schedule dependency of the therapeutic effect of etoposide which has been demonstrated in SCLC, it is important that this is administered over at least 72 hours, and it would not be practical to co-administer amifostine for the full duration of drug exposure.

Another consideration is the use of 740 mg/m$^2$ rather than the more commonly used 910 mg/m$^2$ dose of amifostine in this study. Previous studies have not indicated a significantly different effect at the higher dose level in terms of cytoprotection, and a small crossover trial of cyclophosphamide used with or without amifostine given in a single dose of 740 mg/m$^2$ showed a significant reduction in depth and duration of neutropenia (Glover et al, 1986). There was a suggestion from previous work with amifostine that toxicity was greater at the higher dose, and in the present study there was a significant incidence of side-effects even at 740 mg/m$^2$, with reductions of the total dose administered in 36% of patients for this reason. Whilst it is possible that a higher prescribed dose might have exerted a greater cytoprotective effect, it is also likely that more dose reductions would have been required.

A further factor to consider is whether the chemotherapy was sufficiently myelosuppressive to demonstrate an effect from cytoprotection. Whilst the doses are less than those of the original ICE regimen, a 75% incidence of grade III or IV neutropenia is significant and in keeping with that seen in the previous study of the attenuated ICE regimen. The level of myelosuppression is similar to that seen in the trials of cyclophosphamide and cisplatin for ovarian cancer where a positive effect was demonstrated from the use of amifostine (Glick et al, 1992).

In conclusion, this study has shown that amifostine can be administered safely in combination with modified ICE chemotherapy for SCLC, but that it had no appreciable effect upon the degree of myelosuppression or other toxicities. The contrast with previous studies in which a cytoprotective effect has been shown is most likely to be due to the use of etoposide over a prolonged period. If amifostine is to be used for the attenuation of myelotoxicity in SCLC it will need to be given with drugs all of whose half-life is sufficiently short for the cytoprotective effect to persist for the duration of cytotoxic action. In practice, the use of haemopoietic growth factors may provide a more reliable means to limit neutropenia, and for this reason they are likely to be preferred in the supportive therapy of patients with SCLC undergoing chemotherapy.

### ACKNOWLEDGEMENTS

The following consultants and their collegues participated in the study: Sue Alcock, Dr P B Anderson, Elaine Atkinson, Dr R Banks, Andrew Barker, Perry Blaxill, Dr M G Bond, Chris Button, Ann Camps, Mikhail Cannon, Dr C K Connolly, Dr M Crawford, Deborah Cresswell, Dr M Doherty, Dr M A Greenstone, Emma Guy, Kate Hill, Dr J Hughes, Dr A Hunter, Dr B J Hutchcroft, Dr J Joffe, Professor PWM Johnson, Julia King, Jerry Masterson, Dr D V McGivern, Dr M F Muers, Dr D Parker, Janet Peace, Dr M D Peake, Sue Rodwell, Su Ronaldson, Dorothy Selvey, Dr M Snee, Dr D Spence, Claire Swift, Alex Wakeford, Jenny Ward. We would like to acknowledge the help of Schering Plough in providing the amifostine and of Asta Pharmaceuticals for their support to the Northern and Yorkshire Clinical Trials and Research Unit.

### REFERENCES

Glick J, Kemp G, Rose P et al (1992) A randomized trial of cyclophosphamide and cisplatin + WR-2721 in the treatment of advanced epithelial ovarian cancer. *ASCO abstracts* **11**: 109

Glover D, Glick JH, Weiler C, Hurowitz S and Kligerman MM (1986) WR-2721 protects against the hematologic toxicity of cyclophosphamide: a controlled phase II trial. *J Clin Oncol* **4**: 584–588

Kaplan EL and Meier P (1958) Nonparametric estimation from incomplete observations. *J Am Stat Assoc* **53**: 457–481

Miles DW, Fogarty O, Ash CM et al (1994) Received dose-intensity: a randomized trial of weekly chemotherapy with and without granulocyte colony-stimulating factor in small-cell lung cancer. *J Clin Oncol* **12**: 77–82

Prendiville J, Lorigan P, Hicks F, Leahy B, Stout R, Burt P and Thatcher N (1994) Therapy for small cell lung cancer using carboplatin, ifosfamide, etoposide (without dose reduction), mid-cycle vincristine with thoracic and cranial irradiation. *Eur J Cancer* **14**: 2085–2090

Roth BJ, Johnson DH, Einhorn LH et al (1992) Randomized study of cyclophosphamide plus doxorubicin plus vincristine versus etoposide plus cisplatin versus alternation of these two regimens in extensive small cell lung cancer: A phase III trial of the Southwestern Oncology Group. *Journal of Clinical Oncology* **10**: 282–291

Shevlin PM, Muers MF, Peake MD, Hosker HS, Stead ML, Poulter KM and Brown JM (1998) Modified ice study: a phase II study of an intensive, modified ICE regimen (ifosfamide, carboplatin and etoposide) in patients with better prognosis, small cell lung cancer. *Lung Cancer* **21**: 115–126

Souhami RL and Law K (1990) Longevity in small cell lung cancer. A report to the Lung Cancer Subcommittee of the United Kingdom Coordinating Committee for Cancer Research. *Br J Cancer* **61**: 584–589

Steward WP, von Pawel J, Gatzemeier U, Woll P, Thatcher N, Koschel G, Clancy L, Verweij J, de Wit R, Pfeifer W, Fennelly J, von Eiff M and Frisch J (1998) Effects of granulocyte-macrophage colony-stimulating factor and dose intensification of V-ICE chemotherapy in small-cell lung cancer: a prospective randomized study of 300 patients. *J Clin Oncol* **16**: 642–650

Thatcher N, Girling DJ, Hopwood P, Sambrook RJ, Qian W and Stephens RJ (2000) Improving survival without reducing quality of life in small-cell lung cancer patients by increasing the dose-intensity of chemotherapy with granulocyte

*© 2001 Cancer Research Campaign*

colony-stimulating factor support: results of a British Medical Research Council Multicenter Randomized Trial. Medical Research Council Lung Cancer Working Party. *J Clin Oncol* **18**: 395–404

van der Vijgh WJF and Peters GJ (1994) Protection of normal tissues from the cytotoxic effects of chemotherapy and radiation by amifostine (ethyol): Preclinical aspects. *Seminars in Oncology* **21** (Suppl. 11): 2–7

WHO (1979) Handbook for reporting results of cancer treatments. WHO Offset Publication, No 48,Geneva

Yuhas JM (1980) Active versus passive absorption kinetics as the basis for selective protection of normal tissues by S-2-(3-aminopropylamino)-ethylphosphorothioic acid. *Cancer Research* **40**: 1519–1524

 *© 2001 Cancer Research Campaign*

## References

[PDF_00580] Glover D., Glick J. H., Weiler C., Hurowitz S., Kligerman M. M. (1986). WR-2721 protects against the hematologic toxicity of cyclophosphamide: a controlled phase II trial.. J Clin Oncol.

[PDF_00585] Miles D. W., Fogarty O., Ash C. M., Rudd R. M., Trask C. W., Spiro S. G., Gregory W. M., Ledermann J. A., Souhami R. L., Harper P. G. (1994). Received dose-intensity: a randomized trial of weekly chemotherapy with and without granulocyte colony-stimulating factor in small-cell lung cancer.. J Clin Oncol.

[PDF_00588] Prendiville J., Lorigan P., Hicks F., Leahy B., Stout R., Burt P., Thatcher N. (1994). Therapy for small cell lung cancer using carboplatin, ifosfamide, etoposide (without dose reduction), mid-cycle vincristine with thoracic and cranial irradiation.. Eur J Cancer.

[PDF_00592] Roth B. J., Johnson D. H., Einhorn L. H., Schacter L. P., Cherng N. C., Cohen H. J., Crawford J., Randolph J. A., Goodlow J. L., Broun G. O. (1992). Randomized study of cyclophosphamide, doxorubicin, and vincristine versus etoposide and cisplatin versus alternation of these two regimens in extensive small-cell lung cancer: a phase III trial of the Southeastern Cancer Study Group.. J Clin Oncol.

[PDF_00597] Shevlin P. M., Muers M. F., Peake M. D., Hosker H. S., Stead M. L., Poulter K. M., Brown J. M. (1998). Modified ice study: a phase II study of an intensive, modified ICE regimen (ifosfamide, carboplatin and etoposide) in patients with better prognosis, small cell lung cancer.. Lung Cancer.

[PDF_00601] Souhami R. L., Law K. (1990). Longevity in small cell lung cancer. A report to the Lung Cancer Subcommittee of the United Kingdom Coordinating Committee for Cancer Research.. Br J Cancer.

[PDF_00604] Steward W. P., von Pawel J., Gatzemeier U., Woll P., Thatcher N., Koschel G., Clancy L., Verweij J., de Wit R., Pfeifer W. (1998). Effects of granulocyte-macrophage colony-stimulating factor and dose intensification of V-ICE chemotherapy in small-cell lung cancer: a prospective randomized study of 300 patients.. J Clin Oncol.

[PDF_00609] Thatcher N., Girling D. J., Hopwood P., Sambrook R. J., Qian W., Stephens R. J. (2000). Improving survival without reducing quality of life in small-cell lung cancer patients by increasing the dose-intensity of chemotherapy with granulocyte colony-stimulating factor support: results of a British Medical Research Council Multicenter Randomized Trial. Medical Research Council Lung Cancer Working Party.. J Clin Oncol.

[PDF_00622] Yuhas J. M. (1980). Active versus passive absorption kinetics as the basis for selective protection of normal tissues by S-2-(3-aminopropylamino)-ethylphosphorothioic acid.. Cancer Res.

[PDF_00617] van der Vijgh W. J., Peters G. J. (1994). Protection of normal tissues from the cytotoxic effects of chemotherapy and radiation by amifostine (Ethyol): preclinical aspects.. Semin Oncol.

